# Correspondence

**Published:** 1890-07

**Authors:** W. A. Yingling

**Affiliations:** Nonchalanta, Kansas


### CORRESPONDENCE.

NONCHALANTA, KANSAS, April 2d, 1890.

EDITORS HOMŒOPATHIC PHYSICIAN :—There is *one* thing I wish you would do for the good of all your readers, and *this one* in particular. I am sure this *one* thing would be of highest importance to the great mass of your very large circle of readers. It is this: To publish in THE HOMŒOPATHIC PHYSICIAN a repertory of "*the red string symptoms*," the peculiar characteristic symptoms of the drugs containing them. For instance, Sabina has the *pain from back to pubes* in most complaints. Phos., the *great sense of weakness and emptiness in the abdomen*. Plumbum, *string pulling from abdomen to the back*, etc., etc., etc.

Such a repertory would aid in selecting the proper remedy. I don't mean it to be *the only* symptoms from which to prescribe, but *the* symptom to *lead to the true* remedy covering *all* the case.

Fraternally,

W. A. YINGLING.

[We have such a repertory as above described partly finished. We have no idea when it will be done, as we are already overtaxed with the combined duties of the journal and our practice. Dr. Lee's *Repertory of Characteristics* will be, when it is finished, a complete index to all characteristics, key-notes, and red string symptoms in the materia medica. The second chapter upon the head, for which so much inquiry has been made, is at last nearing completion—five forms, representing eighty pages, having been run through the press.—EDS.]

